# An Open-Source Tool for Anisotropic Radiation Therapy Planning in Neuro-oncology Using DW-MRI Tractography

**DOI:** 10.3389/fonc.2019.00810

**Published:** 2019-08-30

**Authors:** Kesshi Jordan, Olivier Morin, Michael Wahl, Bagrat Amirbekian, Christopher Chapman, Julia Owen, Pratik Mukherjee, Steve Braunstein, Roland Henry

**Affiliations:** ^1^Department of Neurology, University of California, San Francisco, San Francisco, CA, United States; ^2^Joint Graduate Group in Bioengineering, University of California San Francisco and University of California Berkeley, San Francisco/Berkeley, CA, United States; ^3^Department of Radiation Oncology, University of California, San Francisco, San Francisco, CA, United States; ^4^Department of Radiation Oncology, Samaritan Pastega Regional Cancer Center, Corvallis, OR, United States; ^5^Department of Radiology, University of Washington, Seattle, WA, United States; ^6^Department of Radiology and Biomedical Imaging, University of California, San Francisco, San Francisco, CA, United States; ^7^Center for Imaging of Neurodegenerative Disease, San Francisco VA Medical Center, San Francisco, CA, United States

**Keywords:** tractography, glioma, code:Python, neuro oncology, radiation therapy (radiotherapy), diffusion MRI (dMRI)

## Abstract

There is evidence from histopathological studies that glioma tumor cells migrate preferentially along large white matter bundles. If the peritumoral white matter structures can be used to predict the likely trajectory of migrating tumor cells outside of the surgical margin, then this information could be used to inform the delineation of radiation therapy (RT) targets. In theory, an anisotropic expansion that takes large white matter bundle anatomy into account may maximize the chances of treating migrating cancer cells and minimize the amount of brain tissue exposed to high doses of ionizing radiation. Diffusion-weighted MRI (DW-MRI) can be used in combination with fiber tracking algorithms to model the trajectory of large white matter pathways using the direction and magnitude of water movement in tissue. The method presented here is a tool for translating a DW-MRI fiber tracking (tractography) dataset into a white matter path length (WMPL) map that assigns each voxel the shortest distance along a streamline back to a specified region of interest (ROI). We present an open-source WMPL tool, implemented in the package Diffusion Imaging in Python (DIPY), and code to convert the resulting WMPL map to anisotropic contours for RT in a commercial treatment planning system. This proof-of-concept lays the groundwork for future studies to evaluate the clinical value of incorporating tractography modeling into treatment planning.

## Introduction

Tumors which originate from glial cells are known as gliomas, which are the most common form of malignant primary brain tumor. Glioblastoma is the highest grade and most aggressive variant of glioma. The treatment for glioblastoma typically consists of surgery, radiation therapy, and chemotherapy, but the prognosis for patients is still quite poor with a median survival of about 15 months (1).

The standard treatment starts with a maximal safe neurosurgical resection (2). After surgery, the patient typically receives concurrent radiation therapy (RT) and chemotherapy (3). Postmortem investigation has suggested that most recurrences (90%) occur within 2 cm of the primary tumor site (4), so the RT treatment volume (Clinical Target Volume = CTV) is often defined by an isotropic expansion of 1.5–2 cm around the combined tumor bed and any residual tumor (Gross Tumor Volume = GTV). The goal of the CTV expansion is to irradiate infiltrating microscopic disease not visible by standard anatomic imaging in an attempt to reduce tumor recurrence. It should be noted that there is not a consensus on the optimal way to define the CTV. Several studies have tested the accuracy of different RT volume definitions. In a study by Hess et al., the CTV was defined as an isotropic 2 cm expansion, and 86% of recurrences occurred within the treated volume (5). Studies reducing the isotropic expansion to 0.5–1.0 cm around the tumor bed continue to show 80–90% of recurrences within the treated volume (6–8). Another study by Chang et al. investigated whether inclusion of peritumoral edema as defined by T2-weighed MRI abnormality improved target coverage. Whether or not peritumoral edema was included, 10% of patients recurred outside the target volume, although covering peritumoral edema increased the volume of normal brain receiving radiation (9). These studies suggest that for the majority of patients, treatment volumes could be safely reduced without compromising tumor control. Conversely, for a minority of patients with marginal or out-of-field recurrences, further target expansion may have improved tumor control. In both cases, however, isotropic expansion from the tumor bed is likely an oversimplification of the disease biology and recurrence risk pattern.

There is evidence that glioma cells migrate preferentially along large white matter bundles (10–13), so an anisotropic expansion that takes large white matter bundle configuration into account may maximize the chances of radiating migrating cancer cells and minimize the amount of brain tissue radiated. Diffusion-weighted MRI (DW-MRI) can be used in combination with fiber tracking algorithms to model the trajectory of large white matter pathways using the direction and magnitude of water movement in tissue (14–24).

In light of this potential for using DW-MRI to tailor the CTV, several pilot studies have been conducted by researchers seeking to leverage this phenomenon. Berberat et al. found that incorporating diffusion tensor imaging (DTI) into the planning trended toward reduction in CTV volume and still included tumor recurrence sites (25). Kallehauge et al. piloted a DTI driven growth model to similarly adapt the CTV and found that irregular shapes with higher surface area were produced, compared to a more standard CTV, with a dice similarity coefficient of just over 0.7 (26, 27). Jbabdi et al. developed a computational model of brain tumor growth and found that simulation of low-grade glioma growth was improved when anisotropic diffusion was introduced using DTI (28). These studies approach the challenge of leveraging DW-MRI to predict recurrence without a unifying framework to compare and build off of each other's models, indicating that developing a tool for executing these studies would be valuable to the field.

The method presented here is a versatile tool for translating a diffusion MRI (dMRI) fiber tracking (tractography) dataset into a white matter path length (WMPL) map that assigns each voxel the shortest distance along a streamline back to a specified region of interest (ROI). This approach is agnostic to tractography algorithm, enabling the user to test any model that produces streamlines, and can be used to anisotropically modify RT contours. This opens the door for studies that explore models from a simple tensor (DTI) to models that take advantage of high angular resolution diffusion imaging (HARDI) and multi-shell acquisitions. The code was implemented in the Diffusion Imaging in Python (DIPY) package (29) so it interfaces easily with other DIPY tools and can be modified to add new functionality by contributing to this open-source project (dipy.org). This proof-of-concept lays the groundwork for future studies to evaluate the clinical value of incorporating tractography modeling into treatment planning to more specifically target tumor cells that are theoretically migrating along white matter pathways.

## Code

### White Matter Path Length Function

The path_length function (Appendix 1 in Supplementary Material), implemented in python as part of the Diffusion Imaging in Python (DIPY) open-source package (29) (PR 1114) computes the shortest path, along any streamline, between a given region-of-interest (ROI) and each voxel in the specified ROI.

Other supporting functions included in this Pull Request (PR 1114: flexi_tvis_affine, get_flexi_tvis_affine) are used in the path_length function to reconcile affine transformations to map between grid and streamline spaces with different voxel orders. These functions are useful in other applications, like targeting streamlines with an ROI and creating VTK renders.

A demonstration of the path_length function can be found in Appendix 2 in Supplementary Material. The input is a dataset of streamlines in the Trackvis file format (Ruopeng Wang, Van J. Wedeen, TrackVis.org, Martinos Center for Biomedical Imaging, Massachusetts General Hospital) and a region-of-interest in the nifti file format. The mapping from voxel indices to streamline points (affine transformation) is calculated using the function get_flexi_tvis_affine, which inputs the header from the trackvis file and the affine from the nifti file, and outputs a 4 × 4 array. A tutorial is available in the gallery section of the DIPY website (dipy.org).

The output of this white matter path_length function (WMPL map) is an array with each voxel representing the shortest streamline distance back to the given ROI, with voxels not intersected by a streamline set at a filler value (default = −1). The output can be saved in the nifti file format, as demonstrated in Appendix 2 in Supplementary Material. This WMPL map can then be input to a Raystation python script written specifically for translating the path length map to radiation therapy contours in the TPS (Appendix 3 in Supplementary Material),. The WMPL map could be used to generate contours for use with other TP systems, but this would require writing new anisotropic expansion code to translate the WMPL map into contours compatible with other systems.

### Raystation Contour Calculation

The WMPL maps were imported in the treatment planning system (TPS) (RayStation V5.0.2, RaySearch Laboratories, Stockholm, Sweden) in the same frame of reference as the diffusion weighted MR scan. The planning CT scan was rigidly registered to all MR scans (diffusion, T1 post gad, and FLAIR) for creation and transfer of new contours. A python script was created in the TPS to automate the creation of multiple CTVdwMRI ROIs using intensity thresholding of selected expansion path length (EPL) on the WMPL maps. Boolean operations were then applied in order to create CTVdwMRI volumes that excluded any tissue outside the brain. The CTVdwMRI contours consisted of the union of the GTV with the WMPL maps for EPL values of 1, 2, and 3 cm. A 5 mm expansion was finally applied to the results of the union to limit the formation of small contour islands. In total, each patient included in this study had an isotropic 2 cm expansion CTVisotropic around the GTV and 3 anisotropic expansions for each EPL value tested (CTVdwMRI for 1, 2, and 3 cm).

## Methods

### Patients

Thirteen patients with newly diagnosed glioblastoma treated with radiotherapy at the University of California, San Francisco between 2011 and 2015 were selected retrospectively based on the following inclusion criteria. Patients selected were treated with radiotherapy to a dose of 60 Gy in standard fractionation with concurrent temozolomide, underwent High Angular Resolution Diffusion Imaging (HARDI) with the specified parameters (below), underwent regular surveillance MRI (2 month intervals) at UCSF, and had radiographically confirmed recurrence. Nine patients were excluded because they did not have a radiographically confirmed non-disseminated recurrence. All included subjects underwent a HARDI with the following parameters: 55 directions, b = 1,000 s/mm^2^, 1 B0 image, 1.09 × 1.09 mm in-plane resolution, 2 mm slice thickness. Institutional Review Board approval was obtained by the UCSF Committee on Human Research for retrospective analysis of imaging of de-identified patients receiving standard of care for treatment of primary glioblastoma.

### Approach

Given the evidence from pathology studies that glioma cells preferentially migrate along large white matter bundles (10, 11), the use of tractography information to model the trajectory of these pathways should increase the chance that a radiation therapy (RT) treatment volume includes the site of future recurrence. To incorporate the diffusion tractography information into the RT treatment planning software, a path length map was generated to represent the modeled white matter pathway distance. Each voxel was assigned the value of the minimum distance along a streamline connecting it to the Gross Tumor Volume (GTV), defined as the union of the resection cavity and any residual tumor. A radiation oncologist hand-drew each GTV to include the resection cavity and any residual disease, using the T1-weighted post-contrast and T2-weighted FLAIR images.

## Radiation Therapy Planning

### Target Delineation

The GTV was obtained from manual delineation of the post-surgical tumor bed and enhancing regions on post-operative T1-weighted post-contrast MRI. Abnormal mass-like non-enhancing T2-weighted FLAIR signal was also considered for delineation of the GTV. All patients were treated with conventionally defined target volumes, with the clinical target volume generated from 1.5 to 2.0 cm, largely isotropic, expansion of the GTV. A planning target volume (PTV) was generated from 0.3 to 0.5 cm expansion of the CTV. PTV was prescribed 60 Gy with an objective of 95% target coverage. On post-treatment surveillance images showing tumor recurrence/progression, recurrent tumor volumes were also hand-drawn for comparison to original radiation targets.

### Calculation of White Matter Path Length

Residual Bootstrap Q-ball Tractography (30) was performed by seeding all voxels with a fractional anisotropy (FA) >0.15. The tracking was performed at a density of one seed per voxel with the parameters described in Bucci et al. Tracking was terminated by the following stopping criteria: an FA threshold of 0.15 or maximum angle of 60 degrees. The GTV was targeted from this whole-brain streamline dataset to select the subset of streamlines modeling the white matter architecture around the tumor. For visualization purposes, all figures showing GTV-connected streamlines were filtered to remove outliers using the Cluster Confidence Index (CCI > 1; default parameters; implemented in DIPY) (31). The GTV-connected streamlines were converted into a three-dimensional (3D) map, with the value of each voxel representing the minimum white matter path length (WMPL) between the voxel and the GTV. The path length function, implemented in the DIPY open-source package (https://github.com/nipy/dipy/pull/1114), computes the shortest path along any streamline between a given ROI and each voxel in the brain. Each voxel of the WMPL map conveys the shortest distance (in mm) from low to high back to the GTV along a white matter streamline. See Appendix 1 in Supplementary Material for code.

### Creation of the CTV Volumes

The WMPL maps were imported in the treatment planning system (TPS) (RayStation V5.0.2, RaySearch Laboratories, Stockholm, Sweden) in the same frame of reference as the diffusion weighted MR scan. The planning CT scan was rigidly registered to all MR sequences for creation and transfer of new contours. A Python script was written in the TPS to automate the creation of multiple CTVdwMRI volumes using gray-level thresholding of selected expansion path length (EPL) on the WMPL maps. Boolean operations were then applied in order to create CTVdwMRI volumes that excluded any tissue outside the brain. The CTVdwMRI contours consisted of the union of the GTV with the WMPL maps for EPL values of 1, 2, and 3 cm. A 5 mm expansion was finally applied to the results of the union to limit the formation of small contour islands. In total, each patient included in this study had an isotropic 2 cm expansion CTVisotropic around the GTV and three anisotropic expansions for each EPL value tested (CTVdwMRI for 1, 2, and 3 cm). See Appendix 3 in Supplementary Material for code.

## Results

**Figure 1:**
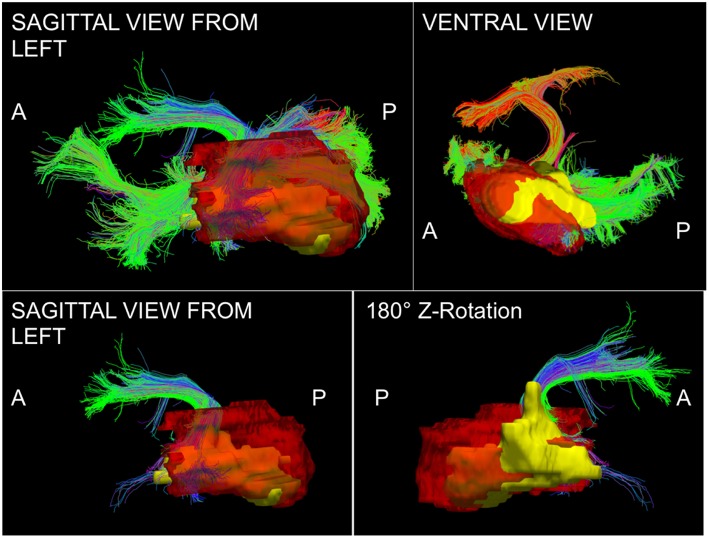
Patient 1 Fiber Tracking Model of GTV White Matter Connectivity: the streamline connectivity of the GTV (red volume) is shown with the recurrence ROI (yellow volume) in the top panels. The bottom panels show the Arcuate Fascicle isolated from the streamline connectivity of the GTV (red volume), it is apparent that the recurrence (yellow volume) follows the path of the bundle modeled using tractography.

**Figure 2:**
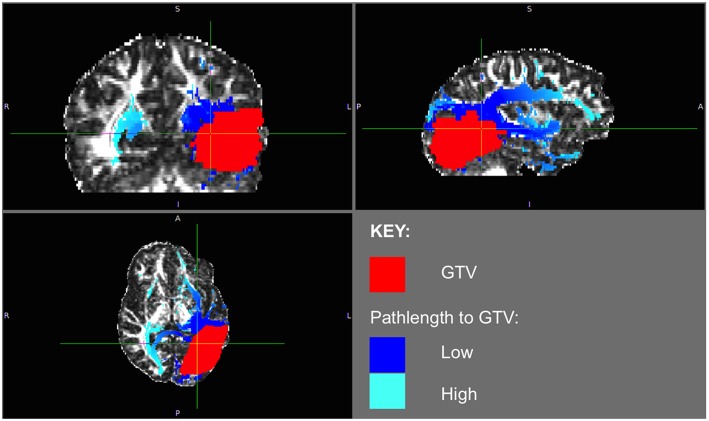
Patient 1 WMPL Map from GTV: The White Matter Path Length (WMPL) map was generated based on the streamline dataset from the GTV. The WMPL shows the distance along streamlines from the GTV from low (dark blue) to high (light blue) distance. The GTV is shown in red.

**Figure 3:**
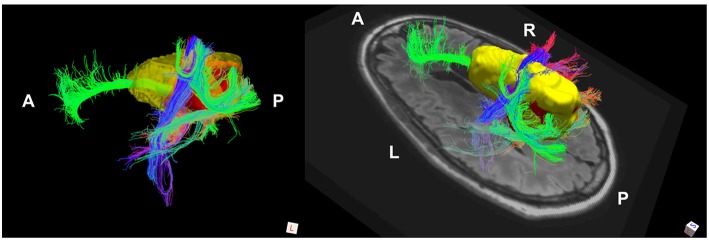
Patient 2 Fiber Tracking Model of GTV White Matter Connectivity: the streamline connectivity of the GTV (red volume) is shown with the recurrence ROI (yellow volume), which appears to follow the path of the cingulum bundle.

**Figure 4:**
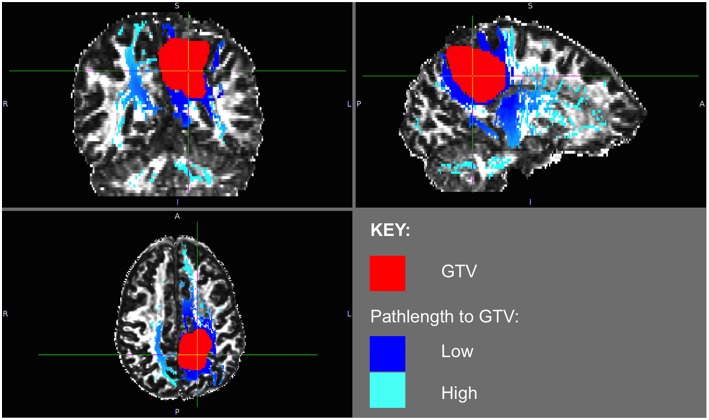
Patient 2 WMPL map from GTV: the White Matter Path Length (WMPL) map was generated based on the streamline dataset from the GTV. The WMPL shows the distance along streamlines from the GTV from low (dark blue) to high (light blue) distance. The GTV is shown in red.

**Figure 5:**
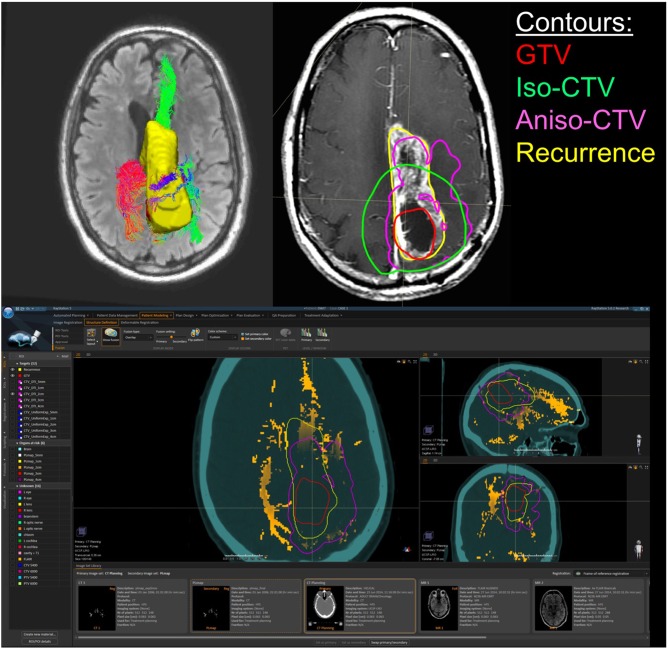
Patient 2 Raystation Treatment Planning Software Anisotropic CTV Based on WMPL Map: this figure (BOTTOM) shows the White Matter Path Length (WMPL) map generated based on the streamline dataset from the GTV and GTV ROI and imported into Raystation (orange). The GTV (red) is expanded anisotropically using the pathlength map (pink), which follows the path of the recurrence (yellow). In the TOP RIGHT figure, the 2 cm anisotropic CTV (pink) follows the path of the recurrence (yellow) better than the 2 cm isotropic CTV (green). In the TOP LEFT figure, the corresponding 3D rendering of the GTV-connected streamlines and the recurrence ROI (yellow) are shown.

The phenomenon of recurrent tumor cells spreading preferentially along white matter pathways was demonstrated by several cases in this cohort. In Patient 1 (Figure 1), the GTV is shown in red with the recurrence shown in yellow. The tractography white matter model of GTV-connected streamlines shows the Arcuate Fascicle, the Inferior Fronto-Occipital Fascicle (IFOF), the Inferior Longitudinal Fascicle (ILF), and the corpus callosum (CC) connected to the GTV. The recurrence appears to be following the trajectory of the Arcuate Fascicle, visible on the 180 degree Z-rotation in Figure 1. The WMPL map (Figure 2) converts the tractogram into a format that can be converted into RT treatment contours. In Patient 2 (Figure 3), the GTV is shown in red with the recurrence shown in yellow. The tractography white matter model of GTV-connected streamlines shows the Cingulum Bundle connected to the GTV. The recurrence appears to be following the trajectory of the Cingulum Bundle. The WMPL map for Patient 2 is shown in Figure 4, and the corresponding anisotropic RT contour planning generated from this WMPL is shown in Figure 5. Code for generating the WMPL map and subsequently anisotropic RT contours in the commercial RT treatment planning system RayStation (RaySearch Laboratories, Stockholm, Sweden) can be found in the Appendix in Supplementary Material.

## Discussion

These WMPL maps can be loaded into an RT planning software to anisotropically modify the treatment volume. An example case is shown in Figure 5. The WMPL map (orange) was used to anisotropically expand the GTV (red) to generate the anisotropic CTV (pink). Compared to the isotropic CTV (green), the anisotropic CTV (pink) better matches the eventual location of recurrence (yellow). This suggests that, in this case, tissue at high risk of subclinical disease could have been predicted by probing the white matter architecture. This procedure was performed on all patients in the pilot cohort that met inclusion criteria to evaluate the clinical application through accuracy and volume of tissue radiated. Results from the retrospective cohort study will be reported elsewhere. Briefly, of 13 cases, three had marginal recurrences using a standard isotropic technique. Using WMPL to define target volumes, two of three marginal recurrences would have been included in the target volume, and all other recurrences would have remained within the target volume. This improved recurrence coverage was accomplished with median 19% smaller target volume than the isotropic technique (range [−34%, +10%]). These pilot results suggest that incorporating white matter microstructure, modeled using dMRI tractography, is a promising approach to predict regions of out-of-field recurrence and reduce tissue at a low risk for subclinical disease. These preliminary results suggest the approach warrants further study to establish sensitivity and specificity measurements in a larger cohort with more marginal and distant recurrences.

The demonstrated evaluation of a diffusion approach is a good method to quickly evaluate whether or not an anisotropic expansion confers added benefit to standard-of-care isotropic expansion. If the anisotropic expansion provided by the underlying streamline maps adds value to the prediction of recurrence, the volume of the anisotropic expansion should be less than that of the isotropic recurrence when both are expanded to the recurrence site in a cohort of patients. After added-value has been established, then a much larger study should be conducted to set expansion parameters so that the methodology can be tested prospectively in a clinical trial.

The flexibility of this approach enables any number of studies to be conducted to investigate the best way to incorporate diffusion information into Radiation Therapy planning. The provided code works on a set of streamlines, so many types of tractography could be investigated, in addition to methods that incorporate human-operator quality-control steps. The target ROI can also be varied to investigate integrating pathway distance from residual tumor, the primary tumor site, or edematous tissue. The timepoint at which the DW-MRI data is collected could also be varied. This pilot was performed using post-surgical pre-radiation therapy HARDI datasets, but pre-surgical HARDI datasets might be more effective to study because the fascicles underlying the tractography have not yet been disturbed by surgical intervention. Many theories of how to use pathway information can be tested with this methodology: for example, large bundles that were cut by the surgery could be isolated to see if neuronal degeneration creates a tumorigenic environment, increasing the chance that the tumor will recur along that pathway. If a set of streamlines can be created to reflect a hypothesis, this framework easily translates the dataset into an added-value analysis to evaluate whether or not that streamline dataset improved prediction of recurrence over isotropic expansion.

## Conclusions

This proof-of-concept demonstrated a tool for integrating diffusion information with existing protocols for Radiation Therapy Planning. The WMPL maps presented in this manuscript can be used to conduct studies predicting tumor recurrence using diffusion data to model white matter architecture. The code to generate the WMPL maps is open-source and freely available on Github as part of the Diffusion Imaging in Python package (29). We have piloted this methodology retrospectively in a cohort of patients that will be reported on elsewhere. We hope that this methodology facilitates investigation of the hypothesis that modifying the CTV based on diffusion models of white matter architecture results in treatment plans that maximize radiation of tissue at high risk for subclinical disease and/or minimize radiation of tissue at low risk for subclinical disease. This proof-of-concept lays the groundwork for future studies to evaluate the clinical value of incorporating tractography modeling into treatment planning to more specifically target tumor cells that are theoretically migrating along white matter pathways.

## Data Availability

The datasets for this study will not be made publicly available because we do not have IRB approval to release these datasets.

## Ethics Statement

IRB was obtained by the UCSF Committee on Human Research for retrospective analysis of imaging of de-identified patients receiving standard of care for treatment of primary glioblastoma.

## Author Contributions

KJ: design, concept/implementation of path length code, data processing and analysis, interpretation, literature research, and manuscript writing. OM: design, concept/implementation of pathlength-to-contour code, data analysis, interpretation, and manuscript writing. MW: concept, design, literature research, clinical information, data processing and analysis, and manuscript writing. BA: implementation of path length code. CC: clinical information, literature research, and critical review. JO: concept, design, data processing, and analysis. PM: concept, design, data collection, and supervision. SB: concept, design, data collection, critical review, and supervision. RH: design, interpretation, critical review, and supervision.

### Conflict of Interest Statement

The authors declare that the research was conducted in the absence of any commercial or financial relationships that could be construed as a potential conflict of interest.
