# Supplementary material for: An Open-Source Tool for Anisotropic Radiation Therapy Planning in Neuro-oncology Using DW-MRI Tractography
Source: Front Oncol. 2019 Aug 30;9:810. doi: 10.3389/fonc.2019.00810 (PMC6730482; doi:10.3389/fonc.2019.00810)
Supplement: Supplementary file 1 [file Data_Sheet_1.pdf]

Appendix 1: Code to Generate White Matter Path Length (WMPL) Map

```
import numpy as np
from dipy.tracking._utils import (_mapping_to_voxel, _to_voxel_coordinates)
from dipy.tracking.utils import _as_segments
from numpy import minimum_at


def path_length(streamlines, aoi, affine, fill_value=-1):
    """ Computes the shortest path, along any streamline, between aoi and
    each voxel.
    Parameters
    ----------
    streamlines : seq of (N, 3) arrays
        A sequence of streamlines, path length is given in mm along the curve
        of the streamline.
    aoi : array, 3d
        A mask (binary array) of voxels from which to start computing distance.
    affine : array (4, 4)
        The mapping from voxel indices to streamline points.
    fill_value : float
        The value of voxel in the path length map that are not connected to the
        aoi.
    Returns
    -------
    plm : array
        Same shape as aoi. The minimum distance between every point and aoi
        along the path of a streamline.
    """
    aoi = np.asarray(aoi, dtype=bool)

    # path length map
    plm = np.empty(aoi.shape, dtype=float)
    plm[:] = np.inf
    lin_T, offset = _mapping_to_voxel(affine, None)
    for sl in streamlines:
        seg_ind = _to_voxel_coordinates(sl, lin_T, offset)
        i, j, k = seg_ind.T
        # Get where streamlines passes through aoi
        breaks = aoi[i, j, k]
        # Where streamline passes aoi, dist is zero
        i, j, k = seg_ind[breaks].T
        plm[i, j, k] = 0

        # If a streamline crosses aoi >1, re-start counting distance for each
        for seg in _as_segments(sl, breaks):
            i, j, k = _to_voxel_coordinates(seg[1:], lin_T, offset).T
            # Get the distance, in mm, between streamline points
            segment_length = np.sqrt(((seg[1:] - seg[:-1]) ** 2).sum(1))
            dist = segment_length.cumsum()
            # Updates path length map with shorter distances
            minimum_at(plm, (i, j, k), dist)
    if fill_value != np.inf:
        plm = np.where(plm == np.inf, fill_value, plm)
    return plm
```

Appendix 2: Example Code to Generate a White Matter Path Length (WMPL) Map

```
from dipy.tracking.utils import get_flexi_tvis_affine, path_length
import nibabel as nib
import os


# Function to save a nifti file
def safesavenii(array, aff, path):
    saveim = nib.Nifti1Image(array, aff)
    saveim.set_qform(aff, 1)
    saveim.set_sform(aff, 1)
    saveim.to_filename(path)


# set the path to the data
basedir = '/path/to/mydata'  # INSERT PATH TO DATA#

# set the path to the ROI (roi) and the streamlines (trk)
roi_pathfrag = 'GTV_diffusion_space.nii.gz'
trk_pathfrag = 'GTV_streamlines.trk'

roipath = os.path.join(basedir, roi_pathfrag)
trkpath = os.path.join(basedir, trk_pathfrag)
savepath = os.path.join(basedir, 'WMPL_map.nii.gz')

# load the streamlines from the trk file
trk, hdr = nib.trackvis.read(trkpath)
sls = [item[0] for item in trk]

# load the ROI from the nifti file
roiim = nib.load(roipath)
roidata = roiim.get_data()
roiaff = roiim.get_affine()

# create mapping between the streamlines and ROI
grid2trk_aff = get_flexi_tvis_affine(hdr, roiaff)

# calculate the WMPL
wmpl = path_length(sls, roidata, grid2trk_aff)

# save the WMPL as a nifti
safesavenii(wmpl, roiaff, savepath)
```

Appendix 3: Code to Generate Radiation Treatment Contours from WMPL Map in RayStation Treatment Planning System

```
"""
GBM dMRI Tractography Script version 2.0.0
Description: Script to expand a GTV volume into an anisotropic CTV using diffusion-wei
ghted MR information processed using the path_length function in Diffusion Imaging in
Python (DIPY) (Kesshi Jordan, UCSF Radiology Pull Request #1114).

Inputs: Minimum path length map (PLmap) produced by DIPY using the GTV as the region o
f interest and a dataset of streamlines modeling white matter tracks of interest (e.g.
tractography seeded from GTV)
Output: CTV contours (Uniform and non-uniform) expanded away from the GTV along white
matter tracks modeled using tractography

Requirements: the planning CT must be named 'CT Planning' & the PLmap 'PLmap'. PLmap a
nd CT Planning are co-registered. GTV and Brain defined on CT Planning.

Running the script:  define the 'PLmap' as primary and the 'CT Planning' as secondary.
Then Run.
Make sure the PLmap and CT Planning are in the same frame of reference.

"""

from connect import *

import math


# #************************************
# Main Program
# #************************************


# Environment setup parameters
db = get_current("PatientDB")
machine_db = get_current("MachineDB")
patient = get_current("Patient")
case = get_current("Case")
examination = get_current("Examination")
structure_set = case.PatientModel.StructureSets[examination.Name]
planning_examination = case.Examinations['CT Planning']
plmap_examination = case.Examinations['PLmap']


case.PatientModel.CopyRoiGeometries(SourceExamination=case.Examinations['CT Planning']
, TargetExaminationNames=["PLmap"], RoiNames=["GTV"])
case.PatientModel.CopyRoiGeometries(SourceExamination=case.Examinations['CT Planning']
, TargetExaminationNames=["PLmap"], RoiNames=["Brain"])

try:
    case.PatientModel.RegionsOfInterest['PLmap_5mm'].DeleteRoi()
    case.PatientModel.RegionsOfInterest['PLmap_1cm'].DeleteRoi()
    case.PatientModel.RegionsOfInterest['PLmap_2cm'].DeleteRoi()
    case.PatientModel.RegionsOfInterest['PLmap_3cm'].DeleteRoi()
    case.PatientModel.RegionsOfInterest['PLmap_4cm'].DeleteRoi()
    case.PatientModel.RegionsOfInterest['CTV_DTI_5mm'].DeleteRoi()
    case.PatientModel.RegionsOfInterest['CTV_DTI_1cm'].DeleteRoi()
```

```
    case.PatientModel.RegionsOfInterest['CTV_DTI_2cm'].DeleteRoi()
    case.PatientModel.RegionsOfInterest['CTV_DTI_3cm'].DeleteRoi()
    case.PatientModel.RegionsOfInterest['CTV_DTI_4cm'].DeleteRoi()
    case.PatientModel.RegionsOfInterest['CTV_UniformExp_5mm'].DeleteRoi()
    case.PatientModel.RegionsOfInterest['CTV_UniformExp_1cm'].DeleteRoi()
    case.PatientModel.RegionsOfInterest['CTV_UniformExp_2cm'].DeleteRoi()
    case.PatientModel.RegionsOfInterest['CTV_UniformExp_3cm'].DeleteRoi()
    case.PatientModel.RegionsOfInterest['CTV_UniformExp_4cm'].DeleteRoi()
except:
    pass

with CompositeAction('Gray level threshold on PLmap image'):

  MyThreshold = -1024

  retval_0 = case.PatientModel.CreateRoi(Name="PLmap_5mm", Color="White", Type="Organ"
, TissueName=None, RoiMaterial=None)
  retval_0.GrayLevelThreshold(Examination=examination, LowThreshold=MyThreshold+2, Hig
hThreshold=MyThreshold+5, PetUnit="", BoundingBox=None)

  retval_0 = case.PatientModel.CreateRoi(Name="PLmap_1cm", Color="Yellow", Type="Organ
", TissueName=None, RoiMaterial=None)
  retval_0.GrayLevelThreshold(Examination=examination, LowThreshold=MyThreshold+5, Hig
hThreshold=MyThreshold+10, PetUnit="", BoundingBox=None)

  retval_0 = case.PatientModel.CreateRoi(Name="PLmap_2cm", Color="Orange", Type="Organ
", TissueName=None, RoiMaterial=None)
  retval_0.GrayLevelThreshold(Examination=examination, LowThreshold=MyThreshold+5, Hig
hThreshold=MyThreshold+20, PetUnit="", BoundingBox=None)

  retval_0 = case.PatientModel.CreateRoi(Name="PLmap_3cm", Color="Red", Type="Organ",
TissueName=None, RoiMaterial=None)
  retval_0.GrayLevelThreshold(Examination=examination, LowThreshold=MyThreshold+5, Hig
hThreshold=MyThreshold+30, PetUnit="", BoundingBox=None)

  retval_0 = case.PatientModel.CreateRoi(Name="PLmap_4cm", Color="Purple", Type="Organ
", TissueName=None, RoiMaterial=None)
  retval_0.GrayLevelThreshold(Examination=examination, LowThreshold=MyThreshold+5, Hig
hThreshold=MyThreshold+40, PetUnit="", BoundingBox=None)

  case.PatientModel.CopyRoiGeometries(SourceExamination=examination, TargetExamination
Names=["CT Planning"], RoiNames=["PLmap_5mm"])
  case.PatientModel.CopyRoiGeometries(SourceExamination=examination, TargetExamination
Names=["CT Planning"], RoiNames=["PLmap_1cm"])
  case.PatientModel.CopyRoiGeometries(SourceExamination=examination, TargetExamination
Names=["CT Planning"], RoiNames=["PLmap_2cm"])
  case.PatientModel.CopyRoiGeometries(SourceExamination=examination, TargetExamination
Names=["CT Planning"], RoiNames=["PLmap_3cm"])
  case.PatientModel.CopyRoiGeometries(SourceExamination=examination, TargetExamination
Names=["CT Planning"], RoiNames=["PLmap_4cm"])

  case.PatientModel.StructureSets['CT Planning'].SimplifyContours(RoiNames=["PLmap_5mm
"], RemoveHoles3D=True, RemoveSmallContours=True, AreaThreshold=1, ReduceMaxNumberOfPo
intsInContours=True, MaxNumberOfPoints=2000, CreateCopyOfRoi=False)
  case.PatientModel.StructureSets['CT Planning'].SimplifyContours(RoiNames=["PLmap_1cm
"], RemoveHoles3D=True, RemoveSmallContours=True, AreaThreshold=1, ReduceMaxNumberOfPo
intsInContours=True, MaxNumberOfPoints=2000, CreateCopyOfRoi=False)
  case.PatientModel.StructureSets['CT Planning'].SimplifyContours(RoiNames=["PLmap_2cm
"], RemoveHoles3D=True, RemoveSmallContours=True, AreaThreshold=1, ReduceMaxNumberOfPo
```

```
intsInContours=True, MaxNumberOfPoints=2000, CreateCopyOfRoi=False)
  case.PatientModel.StructureSets['CT Planning'].SimplifyContours(RoiNames=["PLmap_3cm
"], RemoveHoles3D=True, RemoveSmallContours=True, AreaThreshold=1, ReduceMaxNumberOfPo
intsInContours=True, MaxNumberOfPoints=2000, CreateCopyOfRoi=False)
  case.PatientModel.StructureSets['CT Planning'].SimplifyContours(RoiNames=["PLmap_4cm
"], RemoveHoles3D=True, RemoveSmallContours=True, AreaThreshold=1, ReduceMaxNumberOfPo
intsInContours=True, MaxNumberOfPoints=2000, CreateCopyOfRoi=False)

  # CompositeAction ends

with CompositeAction('ROI Algebra (CTV_Final, Image set: PLmap)'):


  retval_0 = case.PatientModel.CreateRoi(Name="CTV_DTI_5mm", Color="Magenta", Type="Ct
v", TissueName=None, RoiMaterial=None)
  retval_0.SetAlgebraExpression(ExpressionA={ 'Operation': "Union", 'SourceRoiNames':
["GTV"], 'MarginSettings': { 'Type': "Expand", 'Superior': 0, 'Inferior': 0, 'Anterior
': 0, 'Posterior': 0, 'Right': 0, 'Left': 0 } }, ExpressionB={ 'Operation': "Union", '
SourceRoiNames': ["PLmap_5mm"], 'MarginSettings': { 'Type': "Expand", 'Superior': 0, '
Inferior': 0, 'Anterior': 0, 'Posterior': 0, 'Right': 0, 'Left': 0 } }, ResultOperatio
n="Union", ResultMarginSettings={ 'Type': "Expand", 'Superior': 0.5, 'Inferior': 0.5,
'Anterior': 0.5, 'Posterior': 0.5, 'Right': 0.5, 'Left': 0.5 })
  retval_0.UpdateDerivedGeometry(Examination=examination, Algorithm="Auto")
  retval_0.CreateAlgebraGeometry(Examination=examination, Algorithm="Auto", Expression
A={ 'Operation': "Union", 'SourceRoiNames': ["CTV_DTI_5mm"], 'MarginSettings': { 'Type
': "Expand", 'Superior': 0, 'Inferior': 0, 'Anterior': 0, 'Posterior': 0, 'Right': 0,
'Left': 0 } }, ExpressionB={ 'Operation': "Union", 'SourceRoiNames': ["Brain"], 'Margi
nSettings': { 'Type': "Expand", 'Superior': 0, 'Inferior': 0, 'Anterior': 0, 'Posterio
r': 0, 'Right': 0, 'Left': 0 } }, ResultOperation="Intersection", ResultMarginSettings
={ 'Type': "Expand", 'Superior': 0, 'Inferior': 0, 'Anterior': 0, 'Posterior': 0, 'Rig
ht': 0, 'Left': 0 })

  retval_0 = case.PatientModel.CreateRoi(Name="CTV_DTI_1cm", Color="Magenta", Type="Ct
v", TissueName=None, RoiMaterial=None)
  retval_0.SetAlgebraExpression(ExpressionA={ 'Operation': "Union", 'SourceRoiNames':
["GTV"], 'MarginSettings': { 'Type': "Expand", 'Superior': 0, 'Inferior': 0, 'Anterior
': 0, 'Posterior': 0, 'Right': 0, 'Left': 0 } }, ExpressionB={ 'Operation': "Union", '
SourceRoiNames': ["PLmap_1cm"], 'MarginSettings': { 'Type': "Expand", 'Superior': 0, '
Inferior': 0, 'Anterior': 0, 'Posterior': 0, 'Right': 0, 'Left': 0 } }, ResultOperatio
n="Union", ResultMarginSettings={ 'Type': "Expand", 'Superior': 0.5, 'Inferior': 0.5,
'Anterior': 0.5, 'Posterior': 0.5, 'Right': 0.5, 'Left': 0.5 })
  retval_0.UpdateDerivedGeometry(Examination=examination, Algorithm="Auto")
  retval_0.CreateAlgebraGeometry(Examination=examination, Algorithm="Auto", Expression
A={ 'Operation': "Union", 'SourceRoiNames': ["CTV_DTI_1cm"], 'MarginSettings': { 'Type
': "Expand", 'Superior': 0, 'Inferior': 0, 'Anterior': 0, 'Posterior': 0, 'Right': 0,
'Left': 0 } }, ExpressionB={ 'Operation': "Union", 'SourceRoiNames': ["Brain"], 'Margi
nSettings': { 'Type': "Expand", 'Superior': 0, 'Inferior': 0, 'Anterior': 0, 'Posterio
r': 0, 'Right': 0, 'Left': 0 } }, ResultOperation="Intersection", ResultMarginSettings
={ 'Type': "Expand", 'Superior': 0, 'Inferior': 0, 'Anterior': 0, 'Posterior': 0, 'Rig
ht': 0, 'Left': 0 })

  retval_0 = case.PatientModel.CreateRoi(Name="CTV_DTI_2cm", Color="Magenta", Type="Ct
v", TissueName=None, RoiMaterial=None)
  retval_0.SetAlgebraExpression(ExpressionA={ 'Operation': "Union", 'SourceRoiNames':
["GTV"], 'MarginSettings': { 'Type': "Expand", 'Superior': 0, 'Inferior': 0, 'Anterior
': 0, 'Posterior': 0, 'Right': 0, 'Left': 0 } }, ExpressionB={ 'Operation': "Union", '
SourceRoiNames': ["PLmap_2cm"], 'MarginSettings': { 'Type': "Expand", 'Superior': 0, '
Inferior': 0, 'Anterior': 0, 'Posterior': 0, 'Right': 0, 'Left': 0 } }, ResultOperatio
n="Union", ResultMarginSettings={ 'Type': "Expand", 'Superior': 0.5, 'Inferior': 0.5,
```

```
'Anterior': 0.5, 'Posterior': 0.5, 'Right': 0.5, 'Left': 0.5 })
  retval_0.UpdateDerivedGeometry(Examination=examination, Algorithm="Auto")
  retval_0.CreateAlgebraGeometry(Examination=examination, Algorithm="Auto", Expression
A={ 'Operation': "Union", 'SourceRoiNames': ["CTV_DTI_2cm"], 'MarginSettings': { 'Type
': "Expand", 'Superior': 0, 'Inferior': 0, 'Anterior': 0, 'Posterior': 0, 'Right': 0,
'Left': 0 } }, ExpressionB={ 'Operation': "Union", 'SourceRoiNames': ["Brain"], 'Margi
nSettings': { 'Type': "Expand", 'Superior': 0, 'Inferior': 0, 'Anterior': 0, 'Posterio
r': 0, 'Right': 0, 'Left': 0 } }, ResultOperation="Intersection", ResultMarginSettings
={ 'Type': "Expand", 'Superior': 0, 'Inferior': 0, 'Anterior': 0, 'Posterior': 0, 'Rig
ht': 0, 'Left': 0 })

  retval_0 = case.PatientModel.CreateRoi(Name="CTV_DTI_3cm", Color="Magenta", Type="Ct
v", TissueName=None, RoiMaterial=None)
  retval_0.SetAlgebraExpression(ExpressionA={ 'Operation': "Union", 'SourceRoiNames':
["GTV"], 'MarginSettings': { 'Type': "Expand", 'Superior': 0, 'Inferior': 0, 'Anterior
': 0, 'Posterior': 0, 'Right': 0, 'Left': 0 } }, ExpressionB={ 'Operation': "Union", '
SourceRoiNames': ["PLmap_3cm"], 'MarginSettings': { 'Type': "Expand", 'Superior': 0, '
Inferior': 0, 'Anterior': 0, 'Posterior': 0, 'Right': 0, 'Left': 0 } }, ResultOperatio
n="Union", ResultMarginSettings={ 'Type': "Expand", 'Superior': 0.5, 'Inferior': 0.5,
'Anterior': 0.5, 'Posterior': 0.5, 'Right': 0.5, 'Left': 0.5 })
  retval_0.UpdateDerivedGeometry(Examination=examination, Algorithm="Auto")
  retval_0.CreateAlgebraGeometry(Examination=examination, Algorithm="Auto", Expression
A={ 'Operation': "Union", 'SourceRoiNames': ["CTV_DTI_3cm"], 'MarginSettings': { 'Type
': "Expand", 'Superior': 0, 'Inferior': 0, 'Anterior': 0, 'Posterior': 0, 'Right': 0,
'Left': 0 } }, ExpressionB={ 'Operation': "Union", 'SourceRoiNames': ["Brain"], 'Margi
nSettings': { 'Type': "Expand", 'Superior': 0, 'Inferior': 0, 'Anterior': 0, 'Posterio
r': 0, 'Right': 0, 'Left': 0 } }, ResultOperation="Intersection", ResultMarginSettings
={ 'Type': "Expand", 'Superior': 0, 'Inferior': 0, 'Anterior': 0, 'Posterior': 0, 'Rig
ht': 0, 'Left': 0 })

  retval_0 = case.PatientModel.CreateRoi(Name="CTV_DTI_4cm", Color="Magenta", Type="Ct
v", TissueName=None, RoiMaterial=None)
  retval_0.SetAlgebraExpression(ExpressionA={ 'Operation': "Union", 'SourceRoiNames':
["GTV"], 'MarginSettings': { 'Type': "Expand", 'Superior': 0, 'Inferior': 0, 'Anterior
': 0, 'Posterior': 0, 'Right': 0, 'Left': 0 } }, ExpressionB={ 'Operation': "Union", '
SourceRoiNames': ["PLmap_4cm"], 'MarginSettings': { 'Type': "Expand", 'Superior': 0, '
Inferior': 0, 'Anterior': 0, 'Posterior': 0, 'Right': 0, 'Left': 0 } }, ResultOperatio
n="Union", ResultMarginSettings={ 'Type': "Expand", 'Superior': 0.5, 'Inferior': 0.5,
'Anterior': 0.5, 'Posterior': 0.5, 'Right': 0.5, 'Left': 0.5 })
  retval_0.UpdateDerivedGeometry(Examination=examination, Algorithm="Auto")
  retval_0.CreateAlgebraGeometry(Examination=examination, Algorithm="Auto", Expression
A={ 'Operation': "Union", 'SourceRoiNames': ["CTV_DTI_4cm"], 'MarginSettings': { 'Type
': "Expand", 'Superior': 0, 'Inferior': 0, 'Anterior': 0, 'Posterior': 0, 'Right': 0,
'Left': 0 } }, ExpressionB={ 'Operation': "Union", 'SourceRoiNames': ["Brain"], 'Margi
nSettings': { 'Type': "Expand", 'Superior': 0, 'Inferior': 0, 'Anterior': 0, 'Posterio
r': 0, 'Right': 0, 'Left': 0 } }, ResultOperation="Intersection", ResultMarginSettings
={ 'Type': "Expand", 'Superior': 0, 'Inferior': 0, 'Anterior': 0, 'Posterior': 0, 'Rig
ht': 0, 'Left': 0 })


  retval_0 = case.PatientModel.CreateRoi(Name="CTV_UniformExp_5mm", Color="Blue", Type
="Ctv", TissueName=None, RoiMaterial=None)
  retval_0.SetAlgebraExpression(ExpressionA={ 'Operation': "Union", 'SourceRoiNames':
["GTV"], 'MarginSettings': { 'Type': "Expand", 'Superior': 0.5, 'Inferior': 0.5, 'Ante
rior': 0.5, 'Posterior': 0.5, 'Right': 0.5, 'Left': 0.5 } }, ExpressionB={ 'Operation'
: "Union", 'SourceRoiNames': [], 'MarginSettings': { 'Type': "Expand", 'Superior': 0,
'Inferior': 0, 'Anterior': 0, 'Posterior': 0, 'Right': 0, 'Left': 0 } }, ResultOperati
on="None", ResultMarginSettings={ 'Type': "Expand", 'Superior': 0, 'Inferior': 0, 'Ant
erior': 0, 'Posterior': 0, 'Right': 0, 'Left': 0 })
```

```
  retval_0.UpdateDerivedGeometry(Examination=examination, Algorithm="Auto")
  retval_0.CreateAlgebraGeometry(Examination=examination, Algorithm="Auto", Expression
A={ 'Operation': "Union", 'SourceRoiNames': ["CTV_UniformExp_5mm"], 'MarginSettings':
{ 'Type': "Expand", 'Superior': 0, 'Inferior': 0, 'Anterior': 0, 'Posterior': 0, 'Righ
t': 0, 'Left': 0 } }, ExpressionB={ 'Operation': "Union", 'SourceRoiNames': ["Brain"],
'MarginSettings': { 'Type': "Expand", 'Superior': 0, 'Inferior': 0, 'Anterior': 0, 'Po
sterior': 0, 'Right': 0, 'Left': 0 } }, ResultOperation="Intersection", ResultMarginSe
ttings={ 'Type': "Expand", 'Superior': 0, 'Inferior': 0, 'Anterior': 0, 'Posterior': 0
, 'Right': 0, 'Left': 0 })

  retval_0 = case.PatientModel.CreateRoi(Name="CTV_UniformExp_1cm", Color="Blue", Type
="Ctv", TissueName=None, RoiMaterial=None)
  retval_0.SetAlgebraExpression(ExpressionA={ 'Operation': "Union", 'SourceRoiNames':
["GTV"], 'MarginSettings': { 'Type': "Expand", 'Superior': 1, 'Inferior': 1, 'Anterior
': 1, 'Posterior': 1, 'Right': 1, 'Left': 1 } }, ExpressionB={ 'Operation': "Union", '
SourceRoiNames': [], 'MarginSettings': { 'Type': "Expand", 'Superior': 0, 'Inferior':
0, 'Anterior': 0, 'Posterior': 0, 'Right': 0, 'Left': 0 } }, ResultOperation="None", R
esultMarginSettings={ 'Type': "Expand", 'Superior': 0, 'Inferior': 0, 'Anterior': 0, '
Posterior': 0, 'Right': 0, 'Left': 0 })
  retval_0.UpdateDerivedGeometry(Examination=examination, Algorithm="Auto")
  retval_0.CreateAlgebraGeometry(Examination=examination, Algorithm="Auto", Expression
A={ 'Operation': "Union", 'SourceRoiNames': ["CTV_UniformExp_1cm"], 'MarginSettings':
{ 'Type': "Expand", 'Superior': 0, 'Inferior': 0, 'Anterior': 0, 'Posterior': 0, 'Righ
t': 0, 'Left': 0 } }, ExpressionB={ 'Operation': "Union", 'SourceRoiNames': ["Brain"],
'MarginSettings': { 'Type': "Expand", 'Superior': 0, 'Inferior': 0, 'Anterior': 0, 'Po
sterior': 0, 'Right': 0, 'Left': 0 } }, ResultOperation="Intersection", ResultMarginSe
ttings={ 'Type': "Expand", 'Superior': 0, 'Inferior': 0, 'Anterior': 0, 'Posterior': 0
, 'Right': 0, 'Left': 0 })

  retval_0 = case.PatientModel.CreateRoi(Name="CTV_UniformExp_2cm", Color="Blue", Type
="Ctv", TissueName=None, RoiMaterial=None)
  retval_0.SetAlgebraExpression(ExpressionA={ 'Operation': "Union", 'SourceRoiNames':
["GTV"], 'MarginSettings': { 'Type': "Expand", 'Superior': 2, 'Inferior': 2, 'Anterior
': 2, 'Posterior': 2, 'Right': 2, 'Left': 2 } }, ExpressionB={ 'Operation': "Union", '
SourceRoiNames': [], 'MarginSettings': { 'Type': "Expand", 'Superior': 0, 'Inferior':
0, 'Anterior': 0, 'Posterior': 0, 'Right': 0, 'Left': 0 } }, ResultOperation="None", R
esultMarginSettings={ 'Type': "Expand", 'Superior': 0, 'Inferior': 0, 'Anterior': 0, '
Posterior': 0, 'Right': 0, 'Left': 0 })
  retval_0.UpdateDerivedGeometry(Examination=examination, Algorithm="Auto")
  retval_0.CreateAlgebraGeometry(Examination=examination, Algorithm="Auto", Expression
A={ 'Operation': "Union", 'SourceRoiNames': ["CTV_UniformExp_2cm"], 'MarginSettings':
{ 'Type': "Expand", 'Superior': 0, 'Inferior': 0, 'Anterior': 0, 'Posterior': 0, 'Righ
t': 0, 'Left': 0 } }, ExpressionB={ 'Operation': "Union", 'SourceRoiNames': ["Brain"],
'MarginSettings': { 'Type': "Expand", 'Superior': 0, 'Inferior': 0, 'Anterior': 0, 'Po
sterior': 0, 'Right': 0, 'Left': 0 } }, ResultOperation="Intersection", ResultMarginSe
ttings={ 'Type': "Expand", 'Superior': 0, 'Inferior': 0, 'Anterior': 0, 'Posterior': 0
, 'Right': 0, 'Left': 0 })

  retval_0 = case.PatientModel.CreateRoi(Name="CTV_UniformExp_3cm", Color="Blue", Type
="Ctv", TissueName=None, RoiMaterial=None)
  retval_0.SetAlgebraExpression(ExpressionA={ 'Operation': "Union", 'SourceRoiNames':
["GTV"], 'MarginSettings': { 'Type': "Expand", 'Superior': 3, 'Inferior': 3, 'Anterior
': 3, 'Posterior': 3, 'Right': 3, 'Left': 3 } }, ExpressionB={ 'Operation': "Union", '
SourceRoiNames': [], 'MarginSettings': { 'Type': "Expand", 'Superior': 0, 'Inferior':
0, 'Anterior': 0, 'Posterior': 0, 'Right': 0, 'Left': 0 } }, ResultOperation="None", R
esultMarginSettings={ 'Type': "Expand", 'Superior': 0, 'Inferior': 0, 'Anterior': 0, '
Posterior': 0, 'Right': 0, 'Left': 0 })
  retval_0.UpdateDerivedGeometry(Examination=examination, Algorithm="Auto")
  retval_0.CreateAlgebraGeometry(Examination=examination, Algorithm="Auto", Expression
```

```
A={ 'Operation': "Union", 'SourceRoiNames': ["CTV_UniformExp_3cm"], 'MarginSettings':
{ 'Type': "Expand", 'Superior': 0, 'Inferior': 0, 'Anterior': 0, 'Posterior': 0, 'Righ
t': 0, 'Left': 0 } }, ExpressionB={ 'Operation': "Union", 'SourceRoiNames': ["Brain"],
'MarginSettings': { 'Type': "Expand", 'Superior': 0, 'Inferior': 0, 'Anterior': 0, 'Po
sterior': 0, 'Right': 0, 'Left': 0 } }, ResultOperation="Intersection", ResultMarginSe
ttings={ 'Type': "Expand", 'Superior': 0, 'Inferior': 0, 'Anterior': 0, 'Posterior': 0
, 'Right': 0, 'Left': 0 })

  retval_0 = case.PatientModel.CreateRoi(Name="CTV_UniformExp_4cm", Color="Blue", Type
="Ctv", TissueName=None, RoiMaterial=None)
  retval_0.SetAlgebraExpression(ExpressionA={ 'Operation': "Union", 'SourceRoiNames':
["GTV"], 'MarginSettings': { 'Type': "Expand", 'Superior': 4, 'Inferior': 4, 'Anterior
': 4, 'Posterior': 4, 'Right': 4, 'Left': 4 } }, ExpressionB={ 'Operation': "Union", '
SourceRoiNames': [], 'MarginSettings': { 'Type': "Expand", 'Superior': 0, 'Inferior':
0, 'Anterior': 0, 'Posterior': 0, 'Right': 0, 'Left': 0 } }, ResultOperation="None", R
esultMarginSettings={ 'Type': "Expand", 'Superior': 0, 'Inferior': 0, 'Anterior': 0, '
Posterior': 0, 'Right': 0, 'Left': 0 })
  retval_0.UpdateDerivedGeometry(Examination=examination, Algorithm="Auto")
  retval_0.CreateAlgebraGeometry(Examination=examination, Algorithm="Auto", Expression
A={ 'Operation': "Union", 'SourceRoiNames': ["CTV_UniformExp_4cm"], 'MarginSettings':
{ 'Type': "Expand", 'Superior': 0, 'Inferior': 0, 'Anterior': 0, 'Posterior': 0, 'Righ
t': 0, 'Left': 0 } }, ExpressionB={ 'Operation': "Union", 'SourceRoiNames': ["Brain"],
'MarginSettings': { 'Type': "Expand", 'Superior': 0, 'Inferior': 0, 'Anterior': 0, 'Po
sterior': 0, 'Right': 0, 'Left': 0 } }, ResultOperation="Intersection", ResultMarginSe
ttings={ 'Type': "Expand", 'Superior': 0, 'Inferior': 0, 'Anterior': 0, 'Posterior': 0
, 'Right': 0, 'Left': 0 })


  # CompositeAction ends

case.PatientModel.CopyRoiGeometries(SourceExamination=examination, TargetExaminationNa
mes=["CT Planning"], RoiNames=["CTV_DTI_5mm"])
case.PatientModel.CopyRoiGeometries(SourceExamination=examination, TargetExaminationNa
mes=["CT Planning"], RoiNames=["CTV_DTI_1cm"])
case.PatientModel.CopyRoiGeometries(SourceExamination=examination, TargetExaminationNa
mes=["CT Planning"], RoiNames=["CTV_DTI_2cm"])
case.PatientModel.CopyRoiGeometries(SourceExamination=examination, TargetExaminationNa
mes=["CT Planning"], RoiNames=["CTV_DTI_3cm"])
case.PatientModel.CopyRoiGeometries(SourceExamination=examination, TargetExaminationNa
mes=["CT Planning"], RoiNames=["CTV_DTI_4cm"])
case.PatientModel.CopyRoiGeometries(SourceExamination=examination, TargetExaminationNa
mes=["CT Planning"], RoiNames=["CTV_UniformExp_5mm"])
case.PatientModel.CopyRoiGeometries(SourceExamination=examination, TargetExaminationNa
mes=["CT Planning"], RoiNames=["CTV_UniformExp_1cm"])
case.PatientModel.CopyRoiGeometries(SourceExamination=examination, TargetExaminationNa
mes=["CT Planning"], RoiNames=["CTV_UniformExp_2cm"])
case.PatientModel.CopyRoiGeometries(SourceExamination=examination, TargetExaminationNa
mes=["CT Planning"], RoiNames=["CTV_UniformExp_3cm"])
case.PatientModel.CopyRoiGeometries(SourceExamination=examination, TargetExaminationNa
mes=["CT Planning"], RoiNames=["CTV_UniformExp_4cm"])
```
